# Effect of UV-A1 Phototherapy Treatment on Scleroderma: A Systematic Review

**DOI:** 10.7759/cureus.82899

**Published:** 2025-04-24

**Authors:** Stephanie Nagy, Lily Tehrani, Marc M Kesselman

**Affiliations:** 1 Rheumatology, Kiran C. Patel College of Osteopathic Medicine, Nova Southeastern University, Davie, USA

**Keywords:** limited systemic sclerosis, morphea, phototherapy, scleroderma, systemic sclerosis, ultraviolet-a1, uv-a1, uv-a1 phototherapy

## Abstract

Scleroderma is an autoimmune disease characterized by thickened and hardened skin, Raynaud’s phenomenon, calcinosis, telangiectasias, joint and muscle problems, as well as respiratory, cardiac, renal, and gastrointestinal disturbances. Scleroderma can be classified as either localized or systemic. Localized scleroderma refers to sclerosis of isolated areas of the body confined to the skin and subcutaneous layer, but it does not involve the distal extremities. However, an exception to this in some cases is morphea, which may progress to organs. In comparison, systemic scleroderma affects both cutaneous and visceral organs. Diagnosis of the condition is made by clinical presentation, medical history, and diagnostic tests. Current treatment options for scleroderma include cyclophosphamide, methotrexate, azathioprine, mycophenolate mofetil, and hematopoietic stem cell transplant. However, immunosuppressive agents used to treat scleroderma are associated with adverse effects. An unmet need exists for alternative therapies that are tied to a lower risk of adverse events. Ultraviolet (UV)-A1 phototherapy has increasingly been analyzed for use within autoimmune conditions as both a potential first-line or adjuvant treatment option. As such, we conducted a systematic literature review of a total of 293 articles using Ovid, Web of Science, and Cumulative Index to Nursing and Allied Health Literature (CINAHL). Based on our inclusion and exclusion criteria, we included 11 articles in this review, which consisted of a total of 166 patients, the majority being female at 140 (84.3%) patients and only 26 males (15.7%). Overall, patients who received UV-A1 phototherapy saw beneficial effects, including improvements in skin elasticity, mobility of extremities, reduction of skin thickness within sclerotic areas, ulcer improvement, skin softening, reduction of skin tightness, and reduction in collagen bundle size and thickness. UV-A1 phototherapy has the potential to become an integral component of scleroderma management, offering a non-invasive and effective option for patients.

## Introduction and background

Scleroderma, also known as progressive systemic sclerosis, is an autoimmune disorder characterized by skin thickening and hardening, Raynaud’s phenomenon, calcinosis, and telangiectasia. It is additionally associated with musculoskeletal complications, as well as respiratory, cardiac, renal, and gastrointestinal dysfunction. The condition is associated with a high occurrence in the United States, as compared with other countries, with an estimated prevalence rate of 240 per million and an incidence rate of 20 per million per year [1].

**Figure 1:**
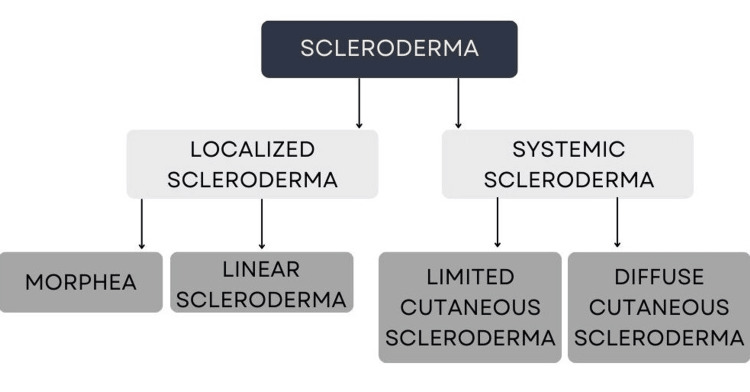
Forms of scleroderma Image Credit: Nagy S, Tehrani L, Kesselman M. This image was created using Canva (https://www.canva.com).

The condition can be classified as either localized or systemic. Localized scleroderma can be further classified into five groups: generalized morphea, plaque morphea, bullous morphea, linear scleroderma, and deep morphea [2]. Systemic scleroderma can also be divided into limited or diffuse sclerosis depending on the clinical and serological criteria (Figure 1). Localized scleroderma refers to sclerosis of isolated areas of the body confined to the skin and subcutaneous layer, but it does not involve the distal extremities. However, an exception to this in some cases is morphea, which may progress to organs, specifically to the central nervous system. In comparison, systemic scleroderma affects both cutaneous and visceral organs. The degree of visceral involvement depends on the patient, but most commonly affects the gastrointestinal tract, lungs, kidneys, skeletal muscle, and pericardium [2, 3]. Limited systemic sclerosis is often identified by its characteristic symptoms: calcinosis, Raynaud’s phenomenon, esophageal dysmotility, sclerodactyly, and telangiectasia (referred to as CREST syndrome). The limited subtype includes skin thickening, which is characteristically distal to the elbows and knees without facial and trunk involvement, while the diffuse subtype is when the sclerosis progresses beyond those areas [2]. Positive antinuclear antibodies have been shown in approximately 90% of patients with systemic sclerosis. In addition, approximately 70% of patients have been shown to present with positive serology for other antibodies, including anti-centromere, anti-Scl-70 (anti-topoisomerase), anti-RNA polymerase III, anti-U1-RNP, and anti-fibrillarin [2, 3].

The pathophysiology of systemic sclerosis is complex and involves a multitude of factors that can lead to progression. At the cellular level, endothelial cell activation and apoptosis are initiated by the deposition of immune complexes containing scleroderma-specific antibodies leading to the activation of toll-like receptors (TLR), amplifying inflammatory pathways, including nuclear factor kappa B (NFκB), p38 mitogen-activated protein kinase (p38MAPK), and stress-activated protein kinases (SAPK)/jun amino-terminal kinases (JNK), as well as upregulating adhesion molecules like intercellular adhesion molecule-1 (ICAM-1) and pro-inflammatory cytokines like interleukin (IL)-6 and IL-8, leading to inflammatory cell recruitment within the vasculature, leading to swelling and lymphohistiocytic infiltrate [4]. In addition to vascular damage, type 2 helper T (Th2) cells become activated upon antigen exposure and produce cytokines such as IL-4 and IL-13, both of which promote fibrosis. Activated myofibroblasts also deposit excess extracellular matrix, leading to thickened and fibrotic skin. Furthermore, B-cells become activated, producing autoantibodies as well as further producing proinflammatory and profibrotic cytokines of IL-6 and transforming growth factor-beta (TGF-B). These B-cells can interact with and activate T-cells to transition into the Th2 subtype and activate fibroblasts into myofibroblasts, as well as trigger autoantibodies to activate endothelial cells, leading to inflammation and cellular damage [5].

Genetic and environmental factors have been demonstrated to potentially play a role in the development and progression of systemic sclerosis. Specifically, a positive family history has been shown to be the strongest predictor of disease onset, as relatives more commonly develop the condition as compared to the general population. Certain HLA haplotypes, including HLA-DRB1, and non-HLA genes like IL-1 and protein tyrosine phosphatase non-receptor 22 gene that are associated with autoimmunity and inflammation, increase the susceptibility to developing scleroderma. Furthermore, connective tissue growth factor and TGF-B have also been found to affect disease severity and susceptibility, as both promote fibrosis activation. Variants in structural and matrix proteins, including fibrillin-1 and secreted protein, which are acidic and rich in cysteine, can lead to altered connective tissue proteins and dysregulation of the extracellular matrix, further promoting fibrotic tissue [5]. In regard to environmental factors, scleroderma has been significantly linked to silica exposure, often found in individuals working in the mining, construction, and sandblasting industries. Additionally, exposure to organic solvents (white spirit, aromatic solvents, chlorinated solvents, trichloroethylene, and ketones) found in the paint and dry cleaning industries and heavy metals (antimony, cadmium, lead, and mercury) has been found to have strong links to scleroderma [6].

Diagnosis of the condition is made by clinical presentation, medical history, and diagnostic tests. There is no definitive treatment or cure for scleroderma; common treatment options for scleroderma include immunosuppressants such as cyclophosphamide, methotrexate, azathioprine, mycophenolate mofetil, and hematopoietic stem cell transplant. Treatment with immunosuppressants can help suppress B and T-cell survival and function but can be associated with adverse effects, including nausea, vomiting, and bone marrow suppression, and can be tied to an increased risk for infections. Biological therapies can also be used for symptom management. For example, monoclonal antibodies, including belimumab, inhibit the B-cell activating factor, leading to reduced levels of IL-4 and IL-10. Meanwhile, most biologics have an increased risk of infections [7]. Glucocorticoids have also been used in the treatment and management of scleroderma, but few studies have been done to determine their efficacy due to the complexity of the disease. As such, an unmet need exists to identify an effective approach that is associated with minimal side effects [8,9].

Ultraviolet (UV)-A1 phototherapy was introduced in the early 1990s as a potential therapy for multiple skin conditions. It uses 340-400 nm radiation energy to penetrate the dermis and target localized cells in the epidermis and dermis [10]. Exposure to UV-A1 causes chromophores, molecules that absorb light, to transfer their energy to oxygen, creating reactive oxygen species (ROS). Singlet oxygen species then attack guanine bases in DNA, induce single-stranded breaks, and promote C-to-T transitions [11]. The creation of ROS can then induce apoptosis (programmed cell death) of lymphocytes, mast cells, and Langerhans cells [12]. UV-A1 also upregulates the enzyme heme oxygenase 1, which has been known to have antifibrotic and antiapoptotic effects. In addition, irradiation exposure has been shown to lead to the inhibition of fibroblasts and the upregulation of matrix metalloproteinases (MMP-1), which helps break down dermal collagen [11]. The anti-inflammatory effects of phototherapy can be attributed to the inhibition of cytokines, which are associated with the Th2 subtype, including IL-5 and IL-13 [13].

The recommendations on the use of UV-A1 phototherapy have yet to be outlined, as organizations are currently debating the efficacy and safety of the treatment of this condition. Currently, the American Academy of Dermatology conditionally recommends UV-A1 phototherapy for atopic dermatitis. In addition, the U.S. Cutaneous Lymphoma Consortium recommends it for mycosis fungoides and Sézary syndrome [14, 15]. The use of UV-A1 for other cutaneous conditions, including subacute prurigo, lichen sclerosus, dyshidrotic dermatitis, urticaria pigmentosa, and pityriasis rosea, requires further research to establish standardized protocols [16].

UV-A1 therapy is currently being evaluated for use in scleroderma, with early evidence demonstrating benefits over current treatment approaches, especially due to the unmet need for more effective and safe approaches. This systematic literature review aims to evaluate current primary studies conducted to gain insight into the use of UV-A1 phototherapy as a potential first-line or adjuvant treatment for patients with all forms of scleroderma.

## Review

Methods

Search Strategy

A systematic literature review was performed using Ovid, Web of Science, and Cumulative Index to Nursing and Allied Health Literature (CINAHL). The search terms used included “scleroderma OR systemic sclerosis OR localized scleroderma” AND “UV-A1 OR ultraviolet-A1 OR ultraviolet-A1 phototherapy OR UV-A1 irradiation therapy.” To ensure the recency of the articles, only articles published between 2010 and 2024 were assessed. The articles were analyzed in a step-wise process by first evaluating the title and abstract for relevancy and then assessing the full-text manuscript. Two authors conducted the first review, and the second-tier reviews were blinded. A third author was utilized to break any conflicts. The Nova Southeastern University (NSU) library database was utilized to access Ovid, Web of Science, and CINAHL and full-text articles.

Selection Criteria

**Figure 2:**
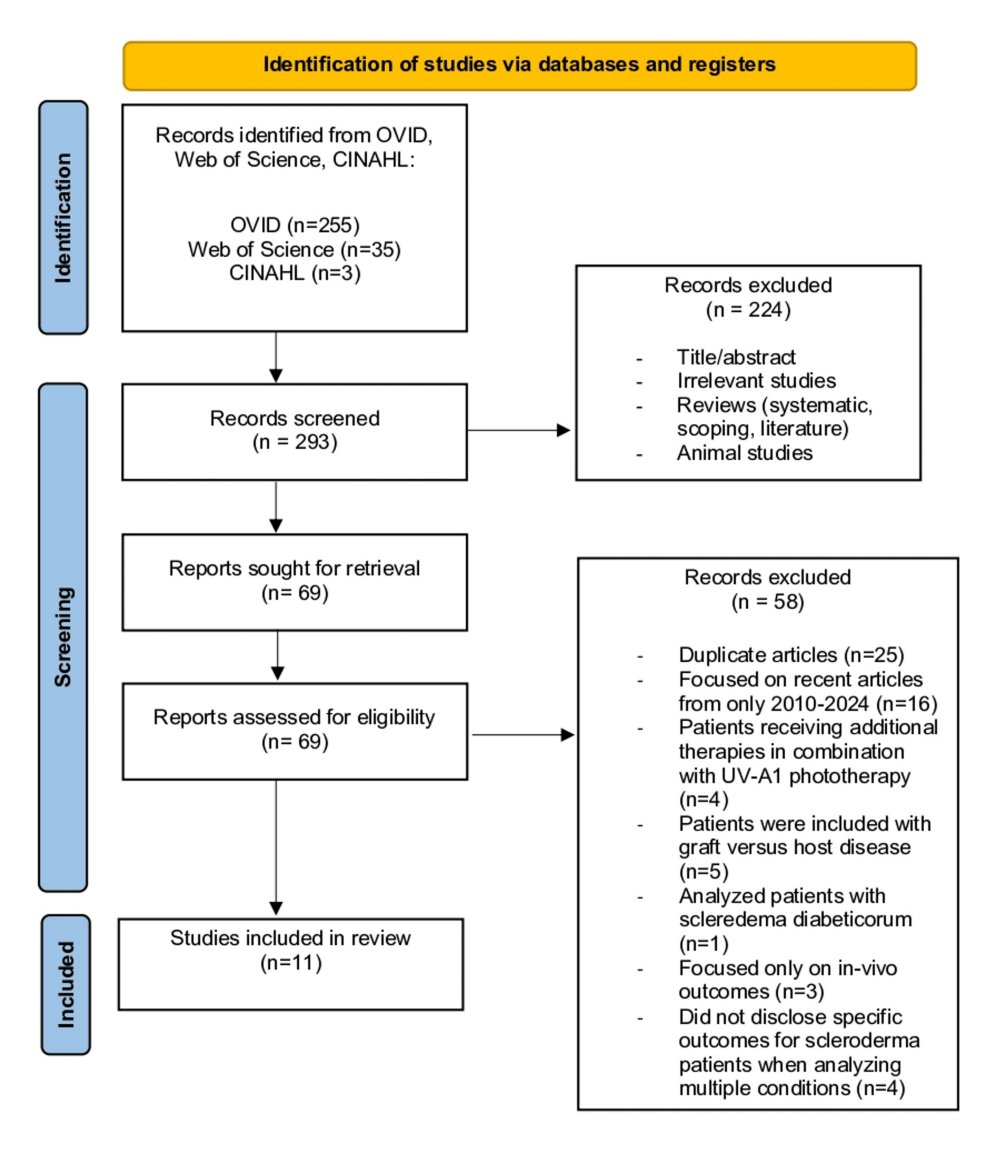
A PRISMA diagram indicating data selection PRISMA: Preferred Reporting Items for Systematic Reviews and Meta-Analyses; CINAHL: Cumulative Index to Nursing and Allied Health Literature

For this review, randomized control trials, cross-sectional studies, observational studies, case reports, and cohort prospective/retrospective studies were included. The population included patients experiencing any form of scleroderma (localized or systemic) as well as their subtypes (morphea, linear, diffuse, or localized forms). Studies excluded from this review were literature, systematic, and scoping reviews, as well as animal studies. Articles were excluded if the patients experienced scleroderma due to graft-versus-host disease, received other forms of treatment for their scleroderma in combination with UV-A1 therapy, or if the article only focused on in vivo outcomes without analyzing the physical changes in order to better understand UV-A1 phototherapy’s potential effects on symptomatology. The Preferred Reporting Items for Systematic Reviews and Meta-Analyses (PRISMA) were used to develop a flow diagram of the selection criteria for reproducibility (Figure 2) [17].

Results

In total, 293 articles were populated between the databases of Ovid, Web of Science, and CINAHL, with 11 final articles included in this review. Table 1 outlines the studies analyzed, detailing the number of patients, sex distribution, average age, scleroderma type, disease duration, symptoms, UV-A1 treatment parameters, treatment duration, and overall outcomes.

**Table 1:** Analyzing the number, age, sex, disease duration, and signs and symptoms of patients as well as the UV-A1 treatment dosage they received, length of treatment, and overall outcomes. M: male; F: female; LoSCT: Localized Scleroderma Cutaneous Assessment Tool

Author	Number of patients	Mean age (years)	Form of scleroderma	Disease Duration (years)	Signs and symptoms of scleroderma	UV-A1 treatment received	Duration of treatment	Outcomes
Ferreira et al. [18]	1 M	44	Diffuse cutaneous systemic sclerosis	8	Esophageal dysmotility, pulmonary fibrosis, microstomia, retraction of the lips, perioral furrows, beaked nose, active finger flexion impairment, restriction on abduction	Initial dose was 10 J/cm^2^, increased and maintained at of 35 J/cm^2^	40 sessions	Greater skin elasticity, improvement in active mobility; improvements were seen within the hands, arms, forearms, anterior chest, and abdomen; Rodnan score decreased from 26 to 11.
Olivet et al. [19]	1 F	47	Systemic sclerosis	12	Taut skin on hands, ulcers on bilateral finger pads	50 J/cm^2^	20 sessions	Improvement in ulcers, skin tightness
Pitney et al. [20]	20 (18 F, 2 M)	49.25	Morphoea (n=12), pansclerotic morphoea (n=3), scleroderma (n=1), systemic sclerosis (n=4)	Not mentioned	Not mentioned.	Initial dose of 30 joules/cm^2^; Average total joules for morphoea group = 2051 J/cm^2^; Average total joules for pansclerotic morphoea group = 5420 J/cm^2^; Average total joules for scleroderma group = 875 J/cm^2^; Average total joules for systemic sclerosis group = 1622 J/cm^2^	72 sessions	Complete response described as greater than 95% clinical clearing and marked softening of skin fibrosis, improved cutaneous elasticity, joint mobility; partial response described as greater than 50% improvement in those same areas; Morphea group = 10 obtained a complete response and 2 obtained a partial response; Pansclerotic Morphea group = 2 obtained a complete response and 1 obtained a partial response; Scleroderma group = 1 obtained a partial response; Systemic sclerosis group = 4 obtained a partial response.
Pereira et al. [21]	20 F	46.48	Plaque-type morphea (n=11), linear morphea (n=3), generalized morphea (n=2), deep morphea (n=1), systemic sclerosis (n=3)	2.5	Plaques, linear, deep lesions, acrosclerosis	Plaque morphea patients = 29 J/cm^2^ dose per session; An average cumulative dose of 1508.5 J/cm^2^; Linear morphea patients = 32.2 J/cm^2^ dose per session, with a average cumulative dose of 2351.7 J/cm^2^; Generalized morphea patients = 32.6 J/cm^2^ dose per session with a average cumulative dose of 1728 J/cm^2^; Deep morphea patients = 34 J/cm^2^ dose per session with a average cumulative dose of 310 J/cm^2^; Systemic scleroderma patients = 29.5 J/cm^2^ dose per session with a average cumulative dose of 1160 J/cm^2^	Morphea patients had an average of 34 sessions; Systemic scleroderma patients had an average of 26 sessions	Patients with morphea, had a marked improvement in 77.8% patients and a moderate improvement was found in 11.1% patients using the Rodnan skin score; Plaque-type morphea had an improvement in the Rodnan score from 7.5 to 2.3 (p<0.003) indicating a 70.8% improvement; Linear-type morphea had an improvement in the Rodnan score from 8 to 2.7 indicating a 66.7% improvement; Generalized morphea had an improvement in the Rodnan score from 16.5 to 9.5 indicating a 40.4% improvement; Deep morphea saw no improvement in Rodnan score; Systemic scleroderma had an improvement in the Rodnan score from 22 to 8 indicating a 63% improvement.
Su et al. [22]	35 (27 F, 8 M)	41.14	Localized scleroderma	5.6	Plaque (n=18), linear (n=4), en coup de sabre (n=3), generalized morphea (n=7), deep or pansclerotic (n=1), and generalized associated with overlying localized scleroderma (n=2) subtypes	Dose of 30 J/cm^2^; Cumulative dose of 1180.2 J/cm^2^	37 sessions	Clinical improvement was observed in 29 of 35 (82. 85%) patients; Ultrasound indicated reduction in skin thickness; Dermal thickness had significant reduction from 3.11 to 2.26 (p<0.002); Sclerosis greatly regressed after fewer than 18 session; All patients showed, softening of the sclerotic lesions resulting in a decreased Rodnan score from 7.91 to 2.85 (p<0.000); Majority of patients reported that treatment had induced a substantial softening of skin lesions; 50% of the lesions disappeared, and more than 50% showed marked improvement during UVA1 irradiation in 29 of the 35 patients. In the remaining 6 patients that did not show benefit, all had late-stage fibrotic white lesions; Sclerotic plaques became smooth and a soft yellowish tanned skin with normal consistency and folding capability; Marked reduction in the highly reflective echo-rich bands within the epidermis and subcutis; Reduction in the thickness of collagen bundles. Collagen bundles appeared to have regular thickness and were separated by regular spaces.
Andres et al. [23]	30 (24 F, 6 M)	56.2	Localized scleroderma	Not reported	Not reported	The cumulative dose ranged between 750-1400 J/cm^2^	20.05 average sessions	Elasticity, inflammation, color, pain, and hardening of lesional plaques improved significantly; Skin elasticity rose from 0.2 mm to 0.3 mm (p=0.04).
Furuhashi et al. [24]	3 F	Case 1= 61, Case 2= 44, Case 3= 36	Case 1 = limited cutaneous systemic sclerosis, Case 2= morphea, Case 3= linear scleroderma	Not reported	Case 1= edema, sclerosis of fingers, Raynaud’s phenomenon, shortened tongue corpuscle, and a mask-like face; Case 2= sclerotic plaque with a lilac ring on her abdomen; Case 3= edema and sclerosis in the right elbow and dorsum of hand, restricted movement of her right wrist and ring finger	Case 1 = 60 J/cm^2^ with a total cumulative of 540 J/cm^2^; Case 2 = 60 J/cm^2^ with a total cumulative of 900 J/cm^2^; Case 3 = 60 J/cm^2^ with a total cumulative of 1800 J/cm^2^	Case 1 = 9 sessions; Case 2 = 15 sessions; Case 3= 30 sessions	Case 1= reduction of skin tightness, collagen bundles decreased in size; Case 2= reduced size of the abdominal sclerotic lesions and attenuated the lilac ring pigmentation; Case 3= Reduced skin tightness of the elbow and dorsal hand lesions. Elastography showed softening of the upper dermis
Connolly et al. [25]	16 (12 F, 4 M)	Not mentioned	Systemic sclerosis	Not reported	Not reported.	5 received low (20–40 J/cm^2^) dose, 6 received medium (>40–80 J/cm^2^) dose, 5 received high (>80–120 J/cm^2^) dose	31 average sessions	Significant association between dosing and improvement for systemic sclerosis with high doses providing the most effectiveness (p = 0.027); Improvements were seen in 20% of those treated with low-dose UV-A1 (n = 5), 83.3% of those treated with medium-dose UV-A1 (n = 6), and 100% of those treated with high-dose UV-A1 (n = 5)
Malewska-Wozniak et al. [26]	5 F	55.7	Morphea	Not reported	Not reported	Dose ranged from 20–50 J/cm^2^	30 average sessions	Reduction in LoSCT (p=0.0001)
Malewska-Wozniak et al. [27]	18 (15 F, 3 M)	50.5	Morphea	1.53	Not reported	Dose ranged from 20–50 J/cm^2^	30 average sessions	Reduction in LoSCT (p=0.0004); Found that the longer the disease duration of morphea, the greater benefits that were seen.
Shalaby et al. [28]	17 (15 F, 2 M)	25.6	Morphea	1.96	Plaque (n=12), linear (n=3), en coup de sabre (n=2)	30 J/cm^2^	30 sessions	Reduction in LoSCT from 6.24 to 4.24 (p=0.001); Collagen homogenization score reduced from 7.53 to 5.41 (p<0.012); Reduction in inflammatory infiltration score from 23 to 14.; Dermal thickness reduced in sclerotic areas from 1.44 mm to 1.06 mm (p<0.017); Non-significant improvement in TGF-B1 score analyzing fibrotic state; Elevated matrix metalloproteinase-1 level indicating increased collagen breakdown in sclerotic areas

**Figure 3:**
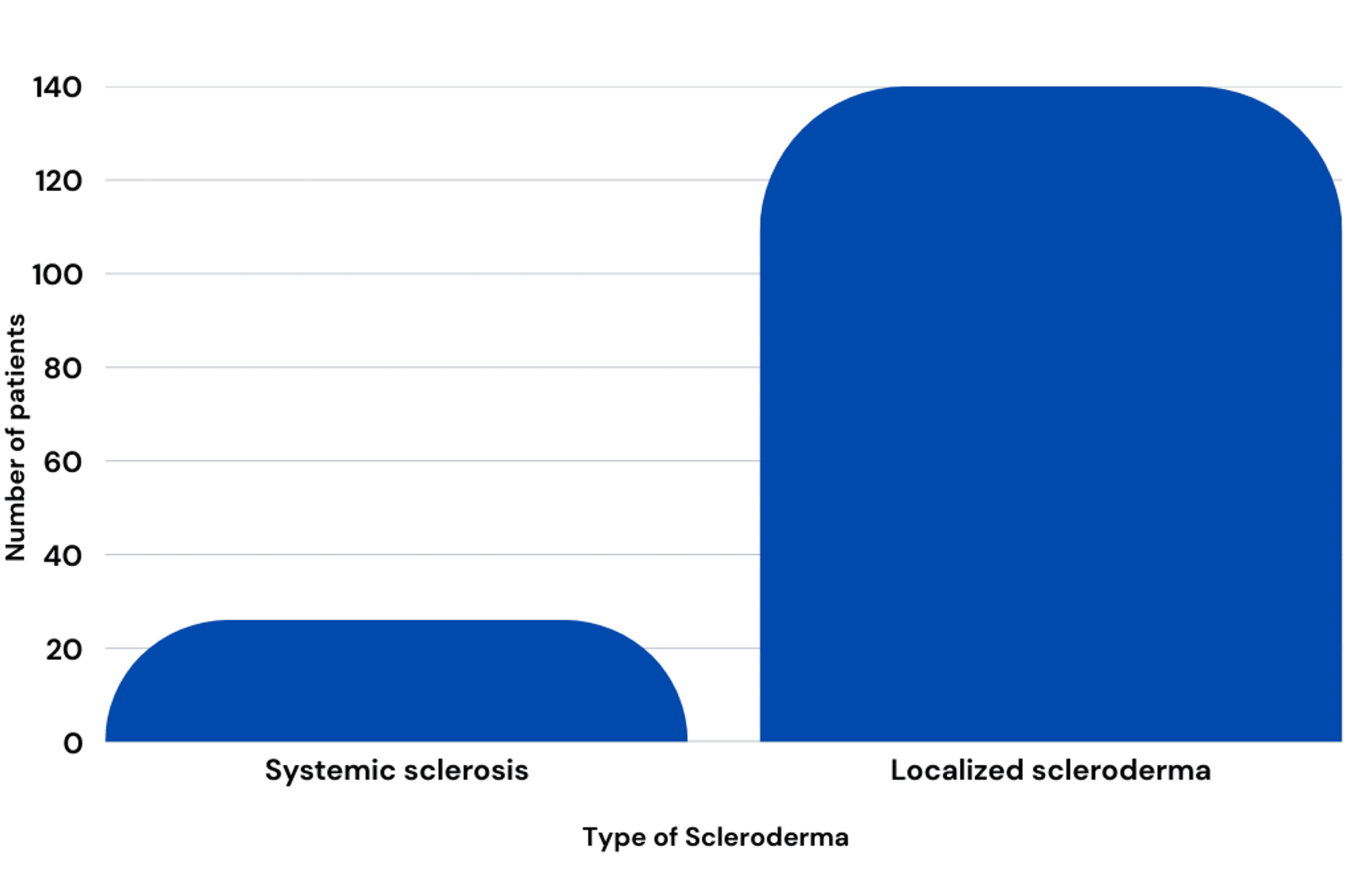
Number of patients diagnosed with systemic and localized scleroderma Image credit: Nagy S, Tehrani L, Kesselman M. This image was created using Canva (https://www.canva.com/).

A total of 166 patients were analyzed, with the majority female (140 (84.3%)) and 26 males (15.7%). The mean age across the studies included was 55.69 years of age. Ten of the 11 studies reported mean age (only Connelly et al. did not report mean age). Scleroderma was separated into two categories: localized or systemic. Subcategories within localized scleroderma included morphea or linear, and subcategories of systemic scleroderma included limited cutaneous or diffuse cutaneous. Patients who were diagnosed with unspecified systemic sclerosis (n=24), and in addition, two patients were specifically categorized as having diffuse cutaneous systemic sclerosis (n=1) or limited cutaneous systemic sclerosis (n=1). Patients were diagnosed with unspecified localized scleroderma (n=65), and some were diagnosed with the subtypes of linear scleroderma (n=1) and morphea (n=74) (including plaque-type morphea, linear morphea, generalized morphea, deep morphea, or pansclerotic morphea). One patient was diagnosed with an unspecified type of scleroderma without indicating systemic or localized type (Figure 3).

Disease duration was reported among seven studies with a mean duration of 5.37 years. The number of treatment sessions among patients ranged from nine to 72 sessions, with a mean of 30 sessions. The dosage of UV-A1 treatment ranged from 10 to 120 J/cm², with a mean of approximately 42.9 J/cm² per session. Low-dose was classified as 20-40 J/cm², medium-dose as 40-80 J/cm², and high-dose as 80-120 J/cm². The majority of patients received low dosages of UV-A1 treatment. Overall, patients who received UV-A1 therapy saw beneficial effects, including improved skin elasticity, mobility of extremities, reduced skin thickness within sclerotic areas, ulcer improvement, skin softening, skin tightness, and reduction in collagen bundle size and thickness.

Discussion

Scleroderma, an autoimmune disease in which the body attacks its connective tissue, has been shown to be associated with inflammation, excess collagen production, and fibrosis. It can be categorized into two major types: localized and systemic. Localized scleroderma affects only the skin, while systemic scleroderma can additionally damage blood vessels and organ systems, including the heart, lungs, and kidneys [29]. While skin manifestations can be treated with immunotherapy and complications of systemic sclerosis can be treated with targeted approaches, treatment can be associated with adverse effects, and a definitive cure has yet to be elucidated. As such, there remains an unmet need for novel therapeutic modalities without a significant risk of adverse effects.

UV-A1 phototherapy is a novel approach that uses non-ionizing radiation, referring to electromagnetic radiation that does not carry enough energy to ionize atoms, to treat a variety of skin conditions. Evaluation of the approach for a variety of dermatologic conditions continues to expand, and early use for a variety of autoimmune conditions, including psoriasis and atopic dermatitis, is growing [30]. UV-A1 is also being explored as a potential therapeutic option for scleroderma patients, especially as converting light to chemical energy can alter cytokine expression [31]. While exposure to UV light has been attributed to the exacerbation of a variety of autoimmune conditions, including systemic lupus erythematosus and dermatomyositis, studies analyzed in this review suggest otherwise: that controlled UV phototherapy at specific wavelengths (340-400 nm) can serve as a therapeutic option for scleroderma [32]. Patients in the analyzed studies had various forms of systemic (n=26) and localized (n=140) scleroderma. Every study reported the benefits of UV-A1 phototherapy, supporting its effectiveness in all types of scleroderma.

**Figure 4:**
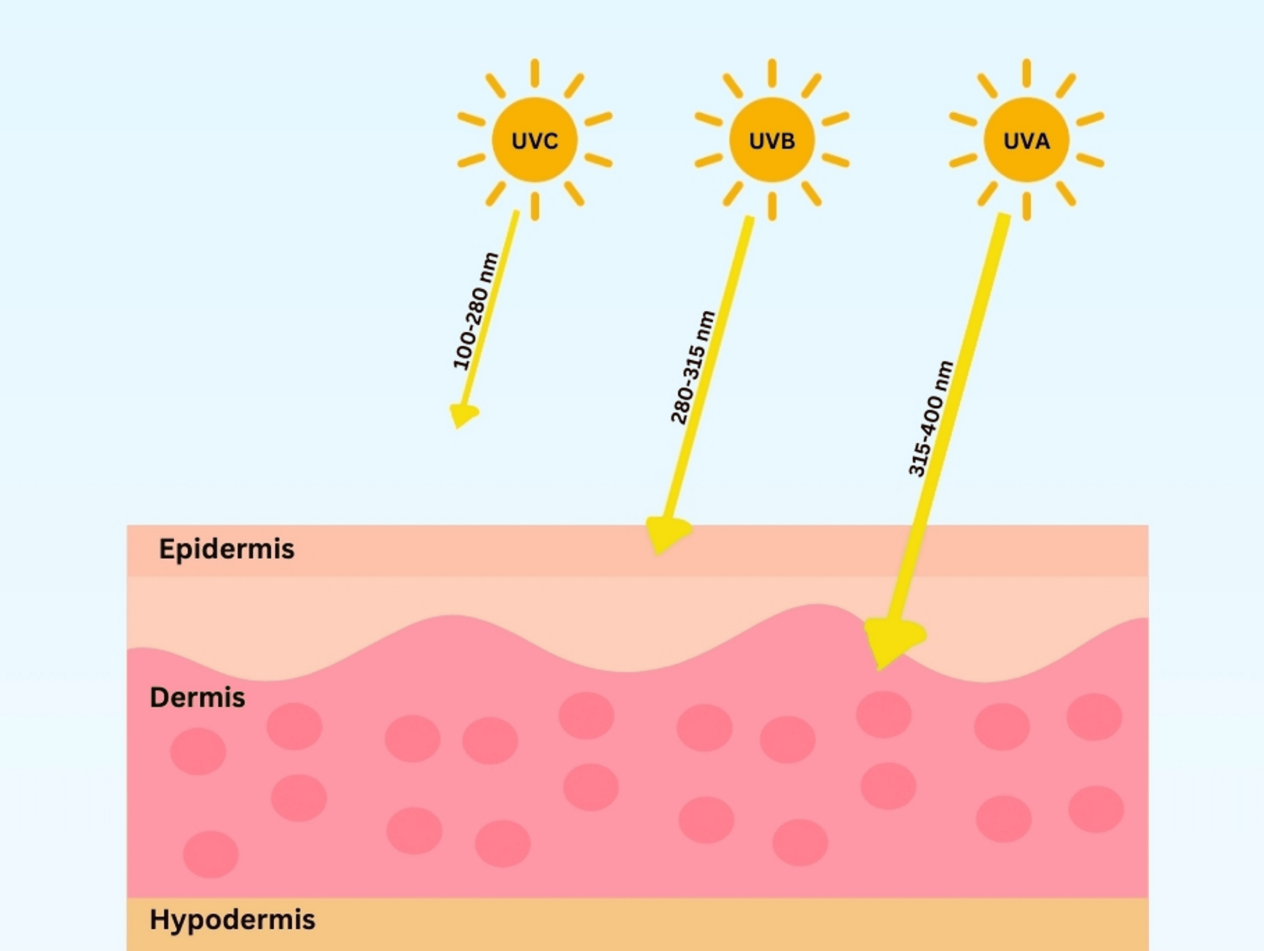
Wavelength pictorial representation of UV-A, UV-B, and UV-C Image credit: Nagy S, Tehrani L, Kesselman M. This image was created using Canva (https://www.canva.com/).

UV radiation can be categorized as UV-C, UV-B, and UV-A. Only UV-B (290-320 nm) and UV-A (UV-A1 340-400 nm and UV-A2 320-340 nm) reach the earth’s surface and penetrate the dermis, while UV-C (200-290 nm) has a limited penetration past the upper dermis layer of skin (Figure 4). As such, only UV-A and UV-B are used in phototherapy. UV-B generally only reaches the epidermis, while UV-A reaches deeper into the dermis layer of skin. Thus, it is more commonly used in therapy. Studies comparing UV-A and UV-B therapy in treating localized scleroderma found significantly more effective results with UV-A1 therapy (p<0.05) [33]. As a result, UV-B has not been identified to date as a beneficial treatment for sclerotic conditions other than in some graft-versus-host diseases.

The majority of the studies analyzed utilized low doses of UV-A1 phototherapy. Meanwhile, previous research efforts have identified that high-dose phototherapy provides superior results and leads to greater softening of plaques [34]. Among the studies analyzed in this review, Connolly et al. was the only one to compare the effectiveness across all three dosing levels, in which the investigators found that higher dosages of UV-A1 phototherapy were 100% effective in treating systemic sclerosis as compared with 83.3% experiencing an improvement with medium doses and 20% with low doses [25]. Connolly et al. did verify a greater efficacy with high dosages of phototherapy; however, all other studies analyzed used low- or medium-dose phototherapy and received equally beneficial results. Interestingly, the American Conference of Governmental Industrial Hygienists and the Canadian Centre for Occupational Health and Safety indicate that individuals exposed to UV-A1 radiation for longer than 1000 seconds should receive only 0.001 J/cm² [35, 36]. Remarkably, within the studies analyzed, the treatment sessions ranged from nine to 72, and the dosage of UV-A1 treatment ranged from 10 to 120 J/cm², with a mean of approximately 42.9 J/cm² per session. These recommendations are to limit the adverse effects that may arise with UV-A1 phototherapy. In the literature, acute toxicity from UV-A1 may result in erythema, edema, blistering, malaise, fever, and nausea, as well as even lead to, in severe cases, photoonycholysis and subungual hemorrhage, while chronic toxicity may be associated with hyperkeratotic papules on non-exposed surfaces, squamous cell carcinoma, and melanoma [30]. However, acute or chronic toxicity was not found within the studies analyzed, even when patients received significantly more than the recommended dose. Only minimal side effects were noted, and the majority found no adverse events in patients. Hyperpigmentation, minimal erythema, and fatigue were the only side effects reported [20, 25, 28].

In addition, it is important to consider the impact of the length of treatment on overall outcomes. Within the studies analyzed, the number of treatment sessions among the patients ranged from nine to 72 sessions, with a mean of 30 sessions. Andres et al. noted positive outcomes tied to both short- and long-term treatments, including a reduction in sclerotic plaques, an increase in skin elasticity, and a reduction of skin thickness [23]. Meanwhile, the negative effects of high dosages and long-term treatment with phototherapy cannot be ignored. As UV-A1 phototherapy continues to expand, consensus on the most effective duration and dose of therapy with limited side effects needs to be elucidated.

The impacts of UV-A1 phototherapy on cellular mechanisms are essential to understanding why UV-A1 therapy leads to these improvements among scleroderma patients. UV-A1 phototherapy has been shown to have multiple in vivo benefits for scleroderma patients. Most notably, the approach has been shown to induce MMP-1 expression, a form of collagenase that degrades collagen I and III bundles, leading to improved skin elasticity and mobility [28,37]. Glutathione levels have also been shown to be reduced with sclerotic lesions, leading to more susceptibility to oxidative damage. Yet, following UV-A1 therapy, a significant elevation of glutathione has been found, indicating elevated protection from reactive oxygen species (ROS) damage [37]. Human β-defensins 1, 2, and 3 are antimicrobial peptides produced by epithelial cells to protect against pathogens and are elevated within the sclerotic plaques. Following UV-A1 phototherapy, human B-defensin-1, -2, and -3 mRNA levels were reduced in lesion skin and unaffected in non-impacted skin. In addition, pro-inflammatory cytokines such as IL-2, IL-4, IL-6, IL-8, and TNF-alpha have also been shown to be pathogenetic in scleroderma, and following phototherapy, pro-inflammatory cytokines were significantly reduced [38]. Meanwhile, in some cases, phototherapy has been found to elevate TNF-alpha within fibroblasts, which plays a key role in reducing collagen bundle thickness by reducing collagen I and III through reducing mRNA levels of collagen and fibronectin and increasing collagenase mRNA in fibroblasts [39]. In addition, it can play a significant role in reducing fibrogenesis by reducing decorin, which is a proteoglycan that is associated with fibrogenesis and TGF-beta reduction within fibroblasts [40, 41]. Furthermore, phototherapy has been shown to induce neovascularization, nourish endothelin cells, and reduce apoptosis, which may lead to replenishment and endothelin transformation [42].

UV-A1 therapy has shown that it may be a beneficial, non-invasive therapy for patients with scleroderma. Scleroderma can significantly impact patients’ quality of life, as the characteristic features of skin thickening, fibrosis, and tightness can lead to restricted mobility and discomfort. The correlation between reduced skin thickness following UV-A1 phototherapy and improved quality of life has been documented across the studies analyzed [18-28]. For example, following therapy, these patients were found to have elevated levels of mobility of hands, arms, forearms, elbows, chest wall, and abdomen due to decreased skin thickness and collagen bundles [18-20, 22-24, 28]. As well as a reduction in ulcers, lessening the risk of infections and morbidity [19]. Furthermore, the appearance of the patient’s cutaneous lesions was reduced in thickness and erythema, leading to softer and smoother lesions, positively impacting self-esteem and reducing conscious worry about the appearance of their skin [20,22,23,28].

Within the studies analyzed, Malewska-Wozniak et al. and Shalaby et al. compared UV-A1 phototherapy to alternative therapies of psoralen plus UV-A (PUVA) and carbon dioxide laser resurfacing in the treatment of scleroderma [27].

PUVA is another form of treatment that has been used for cutaneous conditions, including scleroderma. PUVA combines psoralen, a plant compound that increases skin sensitivity, before receiving UV-A1. Methoxsalen is the most commonly used psoralen. Psoralen compounds intercalate between DNA base pairs following exposure to UV-A1 therapy. They form interstrand crosslinks within the DNA double helix, which reduces DNA synthesis and mitosis, leading to cell apoptosis [43]. Malewska-Wozniak et al. was the only study included in this study to compare PUVA therapy and only UV-A1, finding no statistically significant difference in Localized Scleroderma Cutaneous Assessment Tool (LoSCAT) score, with both resulting in an improvement [27]. Within a large review, no substantial improvements have been found to occur between the use of PUVA versus UV-A1 only, with both having been found to be equally beneficial [44]. With psoralen’s increasing penetration into the tissue, it has been found to elevate the risk of phototoxicity and skin cancers. As such, further evaluation is warranted to weigh the benefits and risks of adding psoralens to UV-A1 therapy [43, 44].

Carbon dioxide laser therapy provides high energy at a wavelength of 10,600 nm with a short pulsation duration of < 1 millisecond. It has been found to produce a heat of up to 66.8°C, which results in collagen contraction leading to skin tightening, as well as collagenases like MMP-1 to degrade excess collagen bundles to reduce skin thickness [45]. Carbon dioxide laser resurfacing has been used to treat scars from acne, trauma, or surgery, as well as photoaging. The use of this technique has been found to have beneficial effects in treating specific symptoms of scleroderma, including the inability to open the mouth, improvement of skin elasticity, and perioral rhytids, as well as reducing dermal atrophy, subcutaneous atrophy, dyspigmentation, and dermal thickness [44, 46, 47]. Shalaby et al. compared the effectiveness of carbon dioxide laser therapy and UV-A1 therapy in morphea patients. The researchers concluded that both methods led to beneficial outcomes for the patients, but there was a greater improvement among those who received the laser therapy [28]. The authors hypothesized that the reason for this improvement was that a greater reduction of TGF-B1 and a greater rise in collagenases MMP-1, broke down the collagen bundles. Meanwhile, this therapy presents with elevated risks as compared to phototherapy due to higher wavelengths and high heat generated, which may lead to deep scarring due to operator error of the laser, hyperpigmentation, contact dermatitis, infection, prolonged erythema, acne, and milia [45]. The investigators were the first to analyze its effectiveness in localized scleroderma. As such, further research is warranted to evaluate carbon dioxide laser therapy within systemic sclerosis to understand its efficacy and safety as compared with UV-A1 phototherapy.

One of the primary limitations of this systematic literature review was the significant variability in UV-A1 phototherapy treatment protocols for scleroderma. Differences in the total joules of UV-A1 radiation delivered, the number of treatment sessions, and the duration between sessions make it challenging to compare study outcomes and to help direct further research toward standardized guidelines. While the short-term benefits of UV-A1 phototherapy have been demonstrated, long-term data on its efficacy and safety remain limited. The potential risks of prolonged UV-A1 exposure, including skin aging, carcinogenesis, and sustained immunomodulatory effects, require further investigation. Standardized study designs with extended follow-up periods will be essential in determining the long-term role of UV-A1 phototherapy in managing scleroderma. In addition, understanding the appropriate patient population who may benefit the most from UV-A1 phototherapy is crucial to providing the best patient-centered care. There is a lack of studies evaluating the effectiveness of UV-A1 phototherapy across different skin types. Given that melanin absorbs UV radiation, skin pigmentation may influence treatment response, potentially affecting both efficacy and the risk of adverse effects. Future research should focus on stratifying patients based on their Fitzpatrick skin type to determine whether specific skin tones respond more favorably to UV-A1 therapy and to refine dosing recommendations accordingly.

Furthermore, it is important to note that approximately 15.2% to 35.8% of patients with systemic sclerosis are positive for anti-SSA antibodies, which have been associated with photosensitivity [48-50]. Future research is required to better understand the tolerance and efficacy of UV-A1 phototherapy for patients positive for anti-SSA antibodies. Currently, caution should be exercised for patients positive for this antibody.

Despite the benefits of UV-A1 phototherapy, optimal dosing, duration, and treatment protocols have yet to be established. This systematic literature review highlights the positive impact of phototherapy within this patient demographic, with the goal of large rheumatologic and dermatologic organizations developing guidelines for the proper use of UV-A1 therapy within different forms of scleroderma.

## Conclusions

UV-A1 phototherapy has emerged as a promising treatment modality for scleroderma. The approach may serve to reduce skin fibrosis, improve elasticity, and modulate immune response among patients with scleroderma. While the exact mechanisms underlying these therapeutic effects continue to be investigated, current evidence supports its role as a viable adjunct therapy. Meanwhile, limitations such as variability in treatment protocols, long-term safety concerns, and patient selection criteria necessitate further large-scale, controlled trials to refine its clinical application. Future research should establish standardized treatment regimens, evaluate combination therapies, and investigate the long-term effects of UV-A1 exposure. With continued advancements, UV-A1 phototherapy has the potential to become an integral component of scleroderma management, offering a non-invasive and effective option for patients.

## References

[1] Mayes MD (2003). Scleroderma epidemiology. Rheum Dis Clin North Am.

[2] Adigun R, Goyal A, Hariz A (2025). Systemic Sclerosis (Scleroderma). https://www.ncbi.nlm.nih.gov/books/NBK430875/.

[3] Careta MF, Romiti R (2015). Localized scleroderma: clinical spectrum and therapeutic update. An Bras Dermatol.

[4] Raschi E, Privitera D, Bodio C (2020). Scleroderma-specific autoantibodies embedded in immune complexes mediate endothelial damage: an early event in the pathogenesis of systemic sclerosis. Arthritis Res Ther.

[5] Thoreau B, Chaigne B, Mouthon L (2022). Role of B-cell in the pathogenesis of systemic sclerosis. Front Immunol.

[6] Marie I, Gehanno JF (2015). Environmental risk factors of systemic sclerosis. Semin Immunopathol.

[7] Katsiari CG, Simopoulou T, Alexiou I, Sakkas LI (2018). Immunotherapy of systemic sclerosis. Hum Vaccin Immunother.

[8] Kowal-Bielecka O, Landewé R, Avouac J (2009). EULAR recommendations for the treatment of systemic sclerosis: a report from the EULAR Scleroderma Trials and Research group (EUSTAR). Ann Rheum Dis.

[9] Iudici M, van der Goes MC, Valentini G, Bijlsma JW (2013). Glucocorticoids in systemic sclerosis: weighing the benefits and risks - a systematic review. Clin Exp Rheumatol.

[10] Calzavara-Pinton P, Bettolini L, Tonon F, Rossi M, Venturini M (2023). The realistic positioning of UVA1 phototherapy after 25 years of clinical experience and the availability of new biologics and small molecules: a retrospective clinical study. Front Med (Lausanne).

[11] Singer S, Berneburg M (2018). Phototherapy. J Dtsch Dermatol Ges.

[12] Godar DE (1999). UVA1 radiation triggers two different final apoptotic pathways. J Invest Dermatol.

[13] Barros NM, Sbroglio LL, Buffara MO, Baka JL, Pessoa AS, Azulay-Abulafia L (2021). Phototherapy. An Bras Dermatol.

[14] Davis DM, Drucker AM, Alikhan A (2024). Guidelines of care for the management of atopic dermatitis in adults with phototherapy and systemic therapies. J Am Acad Dermatol.

[15] Olsen EA, Hodak E, Anderson T, Carter JB, Henderson M, Cooper K, Lim HW (2016). Guidelines for phototherapy of mycosis fungoides and Sézary syndrome: a consensus statement of the United States Cutaneous Lymphoma Consortium. J Am Acad Dermatol.

[16] Ronen S, Ramot Y, Zlotogorski A, Shreberk-Hassidim R (2023). Efficacy of ultraviolet A1 phototherapy for inflammatory, sclerotic and neoplastic dermatological diseases: a 10-year tertiary referral center experience. Photodermatol Photoimmunol Photomed.

[17] Page MJ, McKenzie JE, Bossuyt PM (2021). The PRISMA 2020 statement: an updated guideline for reporting systematic reviews. Syst Rev.

[18] Ferreira BR, Gameiro AR, Brites MM, Oliveira HS, Reis JP (2017). UVA1 for diffuse cutaneous systemic sclerosis in a Fitzpatrick skin type VI patient: outcomes in the modified Rodnan skin score. Acta Reumatol Port.

[19] Olivet MM, Yang K, Graham LV (2024). Improvement of systemic sclerosis-associated digital ulcers after ultraviolet A1 phototherapy. JAAD Case Rep.

[20] Pitney T, Pitney MJ (2020). A retrospective review of UVA1 treatment: an Australian experience from a single centre. Australas J Dermatol.

[21] Pereira N, Santiago F, Oliveira H, Figueiredo A (2012). Low-dose UVA₁ phototherapy for scleroderma: what benefit can we expect?. J Eur Acad Dermatol Venereol.

[22] Su O, Onsun N, Onay HK, Erdemoglu Y, Ozkaya DB, Cebeci F, Somay A (2011). Effectiveness of medium-dose ultraviolet A1 phototherapy in localized scleroderma. Int J Dermatol.

[23] Andres C, Kollmar A, Mempel M, Hein R, Ring J, Eberlein B (2010). Successful ultraviolet A1 phototherapy in the treatment of localized scleroderma: a retrospective and prospective study. Br J Dermatol.

[24] Furuhashi T, Torii K, Ikumi K, Kato H, Nishida E, Morita A (2020). Ultraviolet A1 phototherapy for the treatment of localized scleroderma. J Dermatol.

[25] Connolly KL, Griffith JL, McEvoy M, Lim HW (2015). Ultraviolet A1 phototherapy beyond morphea: experience in 83 patients. Photodermatol Photoimmunol Photomed.

[26] Malewska-Woźniak A, Lodyga M, Adamski Z (2022). Concentrations of metalloproteinase-1 in patients with morphea treated with phototherapy: a preliminary study. Postepy Dermatol Alergol.

[27] Malewska-Woźniak A, Osmola-Mañkowska A, Adamski Z (2022). Effectiveness of PUVA vs. UVA1 phototherapy in the treatment of morphea patients. Postepy Dermatol Alergol.

[28] Shalaby SM, Bosseila M, Fawzy MM, Abdel Halim DM, Sayed SS, Allam RS (2016). Fractional carbon dioxide laser versus low-dose UVA-1 phototherapy for treatment of localized scleroderma: a clinical and immunohistochemical randomized controlled study. Lasers Med Sci.

[29] (2025). Scleroderma. https://www.niams.nih.gov/health-topics/scleroderma.

[30] Rathod DG, Muneer H, Masood S (2025). Phototherapy. https://www.ncbi.nlm.nih.gov/books/NBK563140/.

[31] Hassani J, Feldman SR (2016). Phototherapy in scleroderma. Dermatol Ther (Heidelb).

[32] Estadt SN, Maz MP, Musai J, Kahlenberg JM (2022). Mechanisms of photosensitivity in autoimmunity. J Invest Dermatol.

[33] Kreuter A, Hyun J, Stücker M, Sommer A, Altmeyer P, Gambichler T (2006). A randomized controlled study of low-dose UVA1, medium-dose UVA1, and narrowband UVB phototherapy in the treatment of localized scleroderma. J Am Acad Dermatol.

[34] Stege H, Berneburg M, Humke S (1997). High-dose UVA1 radiation therapy for localized scleroderma. J Am Acad Dermatol.

[35] (2025). International Radiation Protection Association. Radioprotection, du 17 au 22 May.

[36] (2025). Ultraviolet radiation. https://www.ccohs.ca/oshanswers/phys_agents/ultravioletradiation.html.

[37] Yin L, Yamauchi R, Tsuji T, Krutmann J, Morita A (2003). The expression of matrix metalloproteinase-1 mRNA induced by ultraviolet A1 (340-400 nm) is phototherapy relevant to the glutathione (GSH) content in skin fibroblasts of systemic sclerosis. J Dermatol.

[38] Kreuter A, Hyun J, Skrygan M, Sommer A, Bastian A, Altmeyer P, Gambichler T (2006). Ultraviolet A1-induced downregulation of human beta-defensins and interleukin-6 and interleukin-8 correlates with clinical improvement in localized scleroderma. Br J Dermatol.

[39] Takeda K, Hatamochi A, Arakawa M, Ueki H (1993). Effects of tumor necrosis factor-alpha on connective tissue metabolism in normal and scleroderma fibroblast cultures. Arch Dermatol Res.

[40] Sawada H, Isogai Z, Morita A (2003). Altered decorin expression of systemic sclerosis by UVA1 (340-400 nm) phototherapy: immunohistochemical analysis of 3 cases. BMC Dermatol.

[41] Gambichler T, Skrygan M, Tomi NS, Breuksch S, Altmeyer P, Kreuter A (2007). Significant downregulation of transforming growth factor-beta signal transducers in human skin following ultraviolet-A1 irradiation. Br J Dermatol.

[42] Breuckmann F, Stuecker M, Altmeyer P, Kreuter A (2004). Modulation of endothelial dysfunction and apoptosis: UVA1-mediated skin improvement in systemic sclerosis. Arch Dermatol Res.

[43] Richard E (2025). Psoralen plus ultraviolet A photochemotherapy. https://www.uptodate.com/contents/psoralen-plus-ultraviolet-a-puva-photochemotherapy.

[44] Brenner M, Herzinger T, Berking C, Plewig G, Degitz K (2005). Phototherapy and photochemotherapy of sclerosing skin diseases. Photodermatol Photoimmunol Photomed.

[45] Ramsdell WM (2012). Fractional carbon dioxide laser resurfacing. Semin Plast Surg.

[46] Klimek P, Placek W, Owczarczyk-Saczonek A (2022). Fractional ablative carbon dioxide lasers for the treatment of morphea: a case series and literature review. Int J Environ Res Public Health.

[47] Apfelberg DB, Varga J, Greenbaum SS (1998). Carbon dioxide laser resurfacing of peri-oral rhytids in scleroderma patients. Dermatol Surg.

[48] Żebryk P, Przymuszała P, Nowak JK, Piorunek T, Mularek-Kubzdela T, Puszczewicz M (2023). Autoantibodies and clinical correlations in Polish systemic sclerosis patients: a cross-sectional study. J Clin Med.

[49] Burja B, Boubaya M, Bruni C (2024). Anti-Ro/SSA antibodies are predictive of a more severe lung involvement in patients with systemic sclerosis: a study from the heustar database. Ann Rheum Dis.

[50] Sánchez-Montalvá A, Fernández-Luque A, Simeón CP, Fonollosa-Plà V, Marín A, Guillén A, Vilardell M (2014). Anti-SSA/Ro52 autoantibodies in scleroderma: results of an observational, cross-sectional study. Clin Exp Rheumatol.

